# Aging Effect on Audiovisual Integrative Processing in Spatial Discrimination Task

**DOI:** 10.3389/fnagi.2017.00374

**Published:** 2017-11-14

**Authors:** Zhi Zou, Bolton K. H. Chau, Kin-Hung Ting, Chetwyn C. H. Chan

**Affiliations:** Applied Cognitive Neuroscience Laboratory, Department of Rehabilitation Sciences, The Hong Kong Polytechnic University, Kowloon, Hong Kong

**Keywords:** aging, ERP, spatial discrimination, sensory integration, multisensory

## Abstract

Multisensory integration is an essential process that people employ daily, from conversing in social gatherings to navigating the nearby environment. The aim of this study was to investigate the impact of aging on modulating multisensory integrative processes using event-related potential (ERP), and the validity of the study was improved by including “noise” in the contrast conditions. Older and younger participants were involved in perceiving visual and/or auditory stimuli that contained spatial information. The participants responded by indicating the spatial direction (far vs. near and left vs. right) conveyed in the stimuli using different wrist movements. electroencephalograms (EEGs) were captured in each task trial, along with the accuracy and reaction time of the participants’ motor responses. Older participants showed a greater extent of behavioral improvements in the multisensory (as opposed to unisensory) condition compared to their younger counterparts. Older participants were found to have fronto-centrally distributed super-additive P2, which was not the case for the younger participants. The P2 amplitude difference between the multisensory condition and the sum of the unisensory conditions was found to correlate significantly with performance on spatial discrimination. The results indicated that the age-related effect modulated the integrative process in the perceptual and feedback stages, particularly the evaluation of auditory stimuli. Audiovisual (AV) integration may also serve a functional role during spatial-discrimination processes to compensate for the compromised attention function caused by aging.

## Introduction

Multisensory integration occurs when information from different sensory modalities is perceived and synthesized (Stein and Stanford, 2008). Previous studies revealed consistent findings that individual behavioral performances can be enhanced by presenting information to different sensory modalities (Calvert et al., 2001; Corneil et al., 2002; Klucharev et al., 2003; Besle et al., 2004; van Wassenhove et al., 2005; Van Wanrooij et al., 2009). For instance, speech discrimination was improved when messages were presented in the forms of both auditory (spoken words) and visual (lip movement) stimuli (Calvert et al., 2001; Klucharev et al., 2003; Besle et al., 2004; van Wassenhove et al., 2005). The simultaneous presentation of auditory and visual stimuli has also been shown to improve accuracy rates and reaction times in target discrimination or spatial localization tasks (Corneil et al., 2002; Van Wanrooij et al., 2009). Studies using event-related potential (ERP) revealed an early process and a late process associated with audiovisual (AV) integration. The early process is characterized by a C1 wave (~60 to 95 ms) elicited over the parieto-occipital region, reflecting the perception of a visual stimulus. The amplitude of the C1 wave was found to be less positive-going in multisensory condition compared to unisensory conditions. This suggests that multisensory integration involves early perceptual processes in the primary visual and auditory cortices (Cappe et al., 2010). Other studies reported that the integration of audial and visual cues modulated the visuospatial discrimination process (Santangelo and Spence, 2007; Santangelo et al., 2008a,b). The effects were associated with a more positive-going P1 wave (130–150 ms after cue onset) elicited over parieto-occipital sites by the multisensory cues compared to the unisensory cues (Santangelo et al., 2008b). The late process is characterized with a P2 component (around 200 ms post-stimulus) elicited in the frontal and occipital regions (Vidal et al., 2008; Stekelenburg et al., 2013). Compared to unisensory stimuli, multisensory stimuli modulated amplitudes of the P2 at the frontal-central (auditory) and occipital (visual) regions. Earlier studies suggested that fronto-central P2 was sensitive to the physical properties of a sound, such as loudness (Hegerl and Juckel, 1993) and pitch (Novak et al., 1992), as well as spatial location of the sound source in the azimuthal plane (Tiitinen et al., 2006). P2 also was identified as a marker for reflecting attention deficit, such as sensory gating that inhibits the processing of unrelated information (Melara et al., 2002; Crowley and Colrain, 2004; Čeponienė et al., 2005; Barry et al., 2009; Lijffijt et al., 2009; Treder and Blankertz, 2010; Wild-Wall and Falkenstein, 2010). For example, Barry et al. (2009) used a cross-modal paradigm to demonstrate an increase of P2 amplitude among participants with attention deficit when compared to normal controls.

Brain-imaging studies have attributed functional connectivity between the primary visual and auditory cortices to early sensory processing during AV integration (Ghazanfar and Schroeder, 2006; Vetter et al., 2014). Other studies further reported activations in the heteromodal cortices, including most of the association cortices, when synthesizing different sources of unisensory information. The common neural substrates reported in different studies are the superior temporal gyrus (STG) and superior temporal sulcus (Ciaramitaro et al., 2007; Klemen et al., 2009, 2010; Stephen et al., 2010), the inferior parietal sulcus (Saito et al., 2005; Baumann and Greenlee, 2007), the posterior parietal cortex (Amedi et al., 2007; Nardo et al., 2014), and the superior frontal cortex (Baumann and Greenlee, 2007). Specifically, research suggested that the STG mediates the cross AV process, spatial exploration and awareness (Karnath et al., 2001; Stephen et al., 2010).

This study investigates how the aging would modulate AV integration, because the results of most previous studies on neural mechanisms of multisensory integration are based on younger adults. As older adults often experience degenerations in sensory discrimination (Ryan et al., 2012; Ritchie et al., 2014), attention (Jennings et al., 2007; Mahoney et al., 2011), and spatial localization functions (Dobreva et al., 2011; Freigang et al., 2015), it is important to gain an understanding of how these factors would influence AV integration among this population.

Of the few studies on older adults, a majority reported that older participants had greater improvements in their reaction times than younger participants did when responding to AV stimuli (Laurienti et al., 2006; Peiffer et al., 2007; Diederich et al., 2008; Hugenschmidt et al., 2009). However, Stephen et al. (2010) reported contradictory findings that older participants did not show improved reaction times in the AV condition. They further reported age-related differences in the magnetoencephalography amplitude captured at STG around 200 ms. Specifically, the amplitudes captured in the AV condition were significantly lower than those in the auditory-alone (A) condition among the older group; this was described as sub-additive (AV < A). In contrast, the reverse condition was revealed in the younger group, and described as super-additive (AV > A). The sub-additive pattern in the older group was correlated with slower reaction times in the AV condition. These inconsistent findings on the aging effect further motivated the current study. P2 is an important marker in AV integration in this study; its latency was revealed to be significantly delayed in older adults compared to younger adults (Martin and Jerger, 2005; Ross et al., 2007; Ozmeral et al., 2016). This also supports the need for this study.

We are interested in the aging effect on modulating the neural processes associated with multisensory integration. The sensory modalities employed were visual and auditory stimuli that conveyed visuospatial information. The participants performed a spatial discrimination task based on AV (multisensory), visual (V; unisensory), or auditory (A; unisensory) stimuli. Differing levels of noise were added to the visual and auditory stimuli used in this study for the younger and older groups. The difficulty levels of the stimuli, however, were largely matched by having both groups to complete training sessions and reach performance standards set for the tasks before the actual experiment. Instead of employing [AV-V] or [AV-A] to compare the multisensory vs. unisensory conditions in this study, the sum of the unisensory conditions was used to contrast with the multisensory condition. The contrast is expressed as [(AV + C) − (A + V)] (von Saldern and Noppeney, 2013), where C is a neutral condition (containing visual or auditory noise) and (A + V) is the sum of the unisensory conditions (e.g., see Vidal et al., 2008; Cappe et al., 2010). The neutral condition component was proposed (Talsma and Woldorff, 2005; Gondan and Röder, 2006; Mishra et al., 2007) and demonstrated to capture task-general activities, including target processing, response selection and motor processes (Hillyard et al., 1998). It was hypothesized that older participants would have different gains in reaction time compared to the younger participants in the multisensory condition. This aging effect would be reflected by the differences in the P2 component elicited over the fronto-central and occipital areas.

## Materials and Methods

### Participants

The participants were 29 healthy younger adults (16 males, mean age = 24.9 years) and 31 healthy older adults (12 males, mean age = 67.7 years). The younger participants were recruited from local universities, and the older participants were recruited from the local community. All of the participants passed a standard logarithmic visual acuity chart test (>0.8) indicating intact visual acuity ability, and all were able to differentiate the audial stimuli. The exclusion criteria of this study were: (1) receiving musical instrument training (3 months or longer), which may strengthen the connectivity between auditory and motor cortices (Zatorre et al., 2007); and (2) suffering from chronic diseases such as stroke or other neurological disorders. For the older participants, the Hong Kong version of the Montreal Cognitive Assessment (MoCA) was used to screen cognitive dysfunction that might interfere with the multisensory integration process (cut-off ≥ 22). Ethical approval was gained from the institution where the study was conducted (20140627001). This study was carried out in accordance with the human ethic guidelines of The Hong Kong Polytechnic University. Ethics application was submitted to and approved by the Departmental Research Committee of Department of Rehabilitation Sciences. In the application, the information sheet, consent form, and study proposal were vetted by the Committee. Each participant was explained the purpose and procedure of the study. The participant gave written informed consent in accordance with the Declaration of Helsinki.

### Stimuli

Potential differences in the sensitivity of sensory organs between younger and older participants are common in aging-effect studies. The deteriorated sensitivity of older participants was found to bias between-group comparisons of task performances, particularly those involving visual and auditory perception, through a process called inverse effectiveness (Bell et al., 2005; Stanford and Stein, 2007; Stein and Stanford, 2008). Previous studies indicated that use of 100% clear stimuli contributed to inverse effectiveness (Laurienti et al., 2006; Stephen et al., 2010). In this study, noise was added to both the visual and auditory stimuli to increase the difficulty level to 0.75 for both the younger and older groups.

#### Visual Stimuli

**Figure 1:**
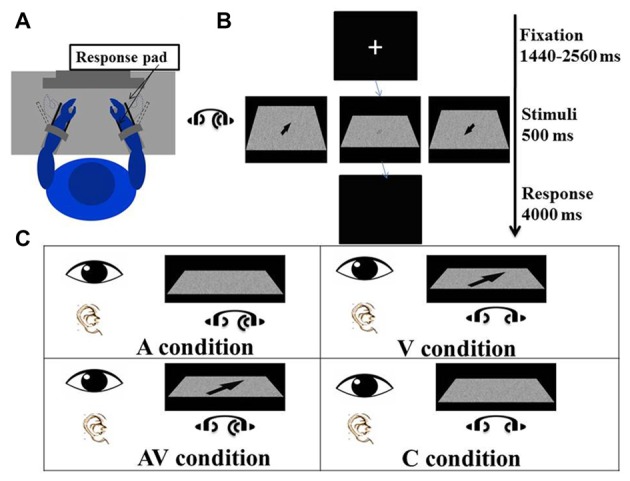
Experimental procedure and conditions. **(A)** The participant sat comfortably in front of a screen with both forearms on the response device on the desk, which had been adjusted to elbow height. Both hands were placed between the two plastic keys (response pads) that were used to give responses. The distance between the eyes and the screen was adjusted to 80 cm. **(B)** The experiment procedure: a fixation cross was first presented at the center of the screen for a random duration between 1440 ms and 2560 ms. Visual and/or auditory stimuli were presented for 500 ms after the fixation. The arrow displayed on the left screen is enlarged to illustrate the direction of a “right far” stimulus; the arrow displayed on the right screen is enlarged to show a “left near” stimulus; and the arrow displayed on the middle screen is a blurred “right far” stimulus as actually seen by the participant. A blank screen was then shown for 4000 ms and participants were asked to indicate the direction of visual and/or auditory stimuli. **(C)** There were four conditions of the experiment in which visual and auditory stimuli were presented simultaneously. In the auditory (A) condition, lateralized “Bat-ears” sound and visual noise were presented. In the visual (V) condition, visual noise with an arrow pointing to one of the four directions and non-lateralized “Bat-ears” sound were presented simultaneously. The audiovisual (AV) condition was composed of visual noise with an arrow and lateralized “Bat-ears” sound. The neutral (C) condition involved visual noise and non-lateralized “Bat-ears” sound.

An arrow was presented in the middle of a 3D space with its head oriented toward one of four locations: left-far (45°), right-far (135°), left-near (315°) and right-near (225°; Figure 1C). The arrow appeared within the foveal region and internal edge = 0.7°, external edge = 1.7°, and center point = 1.2° in the visual field (Bargh and Chartrand, 2000). Gaussian visual noise was added to each arrow image, creating a blurriness effect to the visual stimuli. There were two main reasons for adding the noise to the arrow images: first, participants were required to pay a higher level of attention to blurred images than to clear images in the encoding process, which improved the signal-to-noise ratio of the electroencephalogram (EEG) signals. Second, the comparable difficulty levels in encoding between the visual and auditory stimuli for spatial localization provided a sound basis for between-condition comparisons. The difficulty level of the visual stimuli (after training) was 0.75 (see below). Photoshop software (version CS3 10.0; Adobe Systems) was employed to fabricate arrows with different levels of blurriness: 0 represents totally blurred, while 100 represents totally clear.

Forty visual stimuli were fabricated, 10 in each of the four direction categories. These stimuli were calibrated prior to the experiment by asking younger and older participants to judge the spatial locations conveyed by stimuli fabricated at different blurriness levels (25–90, based on a pilot study). The blurriness levels of visual stimuli that yielded 75%–90% accuracy ranged from 29 to 60 (mean = 37) for the younger group and 40–90 (mean = 76) for the older group.

#### Auditory Stimuli

The auditory stimuli used were the sounds produced by “Bat ears” previously used in two studies on sound localization processes in individuals with blindness (Chan et al., 2012; Tao et al., 2015). The Bat ears emit ultrasound signals (into the environment), receive their echoes (from surrounding obstacles), and convert them into audible “da-da-da” sounds (via a binaural earphone). The auditory stimuli used in the study were fabricated with Bat ears surrounded by four obstacles, each in a different location: left-far (azimuth 45°, 4 m), right-far (azimuth 135°, 4 m), left-near (azimuth 45°, 1 m) and right-near (azimuth 135°, 1 m). The auditory noise (control) stimuli were fabricated from an obstacle located in the middle position (azimuth of 0°, 1 m). At each location, four stimuli were generated with the obstacles erected at slightly different distances within +15 cm around the location (2600–4900 Hz in pitch, 30–55 dB in intensity).

The difficulty level of the auditory stimuli was about 0.75, based on participants’ completion of a 1-h training. Each participant was to perceive a 1000 ms “Bat-ears” sound via earphone, and then indicate the location from which the sound would have been emitted by pressing the designated key on a response device by wrist extension or flexion. Once a 75% accuracy rate was achieved over 40 trials, the participant was asked to respond to the same sounds at 500 ms duration. Participants who failed to achieve the same accuracy rate before the training ended were excluded from the study.

### Audiovisual Spatial Discrimination Task

A task trial began with a fixation cross appearing in the middle of the screen for an average of 2000 ms (ranging from 1440 ms to 2560 ms). The visual and auditory stimuli were presented simultaneously for 500 ms. The participant was to perceive the spatial information embedded in the stimuli and move his or her wrist to indicate the direction and distance conveyed by the stimulus (Figure 1A). For example, right wrist extension or flexion, respectively, indicated right-far or right-near. The same criteria were applied to the left wrist-movement responses. Extension and flexion movements at the wrists were chosen to produce the responses because it would be easier for the participants to associate the “far” and “near” spatial relationships with these movements than with designated keys on a keyboard. The participants could relate the distance of the wrist “away from the body” and “toward the body” to guide their responses. In contrast, if a keyboard had been used in the study, the participants would have required training to associate “far” and “near” with specific keys. Wrist responses were recorded by a response pad located parallel to the palm and dorsum of both hands (see Procedure below), from which accuracies and reaction times were derived. The time allowed for making a response was 4000 ms (Figure 1B). The mean inter-trial interval was 6220 ms, ranging from 5740 ms to 6660 ms.

There were four conditions—namely visual (V), auditory (A), audiovisual (AV) and control (C). In the V condition, an arrow pointing to one of four directions (right-far, left-far, right-near, and left-near) was presented on the visual noise board, together with auditory noise. The participant was to create a wrist movement based on the direction and location of the arrow. In the A condition, a “Bat-ears” sound that contained lateralized spatial information was presented together with a visual noise board. The participant was to respond to the auditory stimulus. In the AV condition, both visual and auditory stimuli that contained spatial information were presented simultaneously. The participant was to respond by taking into account the spatial information contained in both stimuli. The C condition presented both the visual and auditory noise (Figure 1C), and the participant was to respond by moving the wrist to touch any one of the four response pads. There were 224 trials for each condition, totaling 896 trials. These were divided into eight blocks, with the four-condition trials randomized in each block. The time taken to complete all the blocks was about 2.5 h, including rest periods between the blocks.

### Procedures

Each participant sat on a chair in front of an adjustable-height table located in a dim and soundproof chamber. The participant rested both forearms on the table with elbows flexed at 90°. The forearms and wrists assumed neutral positions and were strapped to the response devices (Figure 1A) to prevent excessive movement in the shoulders and forearms when participants indicated responses with their wrists, hence reducing potential artifacts to the EEG signals. The left and right wrists were aligned with the respective response pads. The response pads were two vertically erected plastic boards (5 cm × 3 cm) located parallel to the dorsum and palm of the hand. The distance between the two plastic boards was adjusted so they were positioned on the palms of the participant. The plastic boards were connected to a computer, and response signals (type and time) were registered when the board was moved by 30° extension or flexion at the wrist. The participant was informed of the task processes involved before one task block began. After making a movement response with the wrist, the participant was instructed to resume a neutral wrist position before the next trial commenced. The participant was reminded to make the response at the wrist as accurately and quickly as possible.

### ERP Data Acquisition and Pre-Processing

An EEG was captured with a 64-channel cap using a NuAmps Digital DC EEG Amplifier and Curry 7 software (Compumedics Neuroscan, USA). The montage was referenced to the right ear lobe electrode. A ground electrode was placed on the forehead. Reference impedances were set below 5 kΩ, and all inter-electrode impedances were maintained below 5 kΩ. Vertical and horizontal electrooculograms (EOGs) were recorded by two pairs of electrodes to monitor eye movements. EEG signals were amplified and digitized at a sampling rate of 1024 Hz.

EEG data preprocessing was conducted using Curry 7, including re-referencing the data to half of the M2 electrode placed at the right mastoid process, with baseline correction of the data using a time window between −200 ms and 0 ms before stimulus presentation. A digital band-pass filter was used with a range of 0.1–30 Hz. The criterion for registering eye movements was set as ±100 μV in both horizontal and vertical EOG channels. EEG data were corrected using the covariance analysis algorithm when eye movement was detected. Epochs were cut from −200 ms to 1000 ms from the onset of each stimulus. Those with an amplitude larger than 100 μV were discarded from the analysis. Only trials with correct responses were further processed.

### Data Analysis

#### Behavioral Analysis

Preliminary review of the data showed a potential speed-accuracy trade-off as there were significant correlations between participants’ accuracy rates and reaction times (A condition: *r* = −0.230, *P* = 0.108; V condition: *r* = −0.338; *P* = 0.016; AV condition: *r* = −0.320, *P* = 0.023). The speed-accuracy trade-off would confound the results if participants had produced slower reaction times to achieve higher accuracy rates, or vice versa. To tackle the speed-accuracy trade off, the inverse efficiency score (IES) method (Townsend and Ashby, 1978)—dividing reaction time by accuracy rate—was used to analyze the behavioral data in the experimental conditions. Behavioral data in the control condition was not analyzed as it did not involve accuracy rate. Two analysis of variance (ANOVA) models were conducted to test the differences between the younger and older participant in terms of performance on the AV, A and V conditions. The first model tested condition-based performance, i.e., IES of AV, A and V; while the second model tested the gain (called modulation, M) in the AV condition compared to the A or V conditions.

In the first model, two-way repeated measures ANOVA was used to test the effects of age (younger or older) and condition (AV, A, or V), as well as their interactions, on the IES. Significant interaction effects were followed by conducting *post hoc* pairwise comparisons among the levels of each factor.

The second model catered to the potential variations in the participants’ task performances due to differences in the sensitivity of their visual and auditory senses. A modulation effect was constructed to further test the task effects. The modulation due to the multisensory over visual condition was defined as the difference in IES between the AV and V condition, i.e., (AV-V). In contrast, the modulation due to the multisensory over auditory condition was defined as the difference in IES between AV and A condition, i.e., (AV-A). This model, therefore, repeated measures ANOVA, testing age and modulation ([AV-V], [AV-A]) effects. Statistical significance was set as *P* < 0.05. IBM SPSS Statistics 23.0 for Windows was used to analyze the data.

#### ERP Waveform Analysis

The multisensory integration effect expressed by [(AV + C) − (A + V)] was tested in terms of the EEG signal amplitudes. The amplitudes were derived for each time point and electrode. There were 300 time points for the 50–350 ms epoch and 60 electrodes (data in CB1 and CB2 not analyzed). Pairwise comparisons using paired *t*-tests were conducted among all the time points for each electrode. Previous articles defined significant between-condition/group differences as differences in >24 consecutive time points (24 ms) from zero (*P* < 0.05; Guthrie and Buchwald, 1991; Stekelenburg and Vroomen, 2007; Vidal et al., 2008). Therefore, a significant AV effect for each channel was defined as when >24 consecutive time points (24 ms) showed significant between-group differences. The employment of the consecutive time-point method has two purposes. First, the procedure is relevant to analyses involving subtraction of signals between two conditions, as was the case in this study, so the results produced would be more robust than those identified based on single or undefined time points within the time window. Second, the robustness of the significant results can reduce Type I errors in the subsequent ANOVA analyses (Guthrie and Buchwald, 1991). Amplitudes significantly larger than zero mean super-additivity due to multisensory integration, while those significantly smaller than zero mean sub-additivity.

The younger and older groups were analyzed separately. To test the aging effect, different time windows were defined for each group. The P2 was defined as the second positive-going wave captured at electrode sites over the fronto-central or parieto-occipital regions. The time window for the younger participants was set at 150–230 ms, based on the waveform observed in this study and the time windows reported in previous studies (Liu et al., 2014; Marsic et al., 2015). The time window for the older participants was set at 190–270 ms (40 ms delay), which was based on the results of previous studies demonstrating a delayed onset latency of P2 due to the aging effect (Martin and Jerger, 2005; Ross et al., 2007; Ozmeral et al., 2016). The peak latencies were verified, and mean amplitudes of the P2 were obtained for each electrode site. *T*-tests were used to compare the between-group differences in the mean amplitudes based on [(AV + C) − (A + V)] at each electrode site. The relationships between the mean amplitudes [(AV + C) − (A + V)] of P2 and the modulation effects of the multisensory integration—i.e., IES (AV-V) and IES (AV-A)—were explored using stepwise multiple regression analysis and whether age was a moderator of this relationship. In the first step, age and amplitudes [(AV + C) − (A + V)] of P2 were the regressors. In the second step, an interaction term (centered age × P2 amplitude) was added to the model (Aiken et al., 1991). To further explore the aging effects, correlations were computed between the amplitudes of P2 yielded in different experimental conditions [(AV + C) − (A + V)] and the subscale and total scores of MoCA for participants in the older group. Statistical significance was set as *P* < 0.05.

## Results

### Behavioral Results

**Figure 2:**
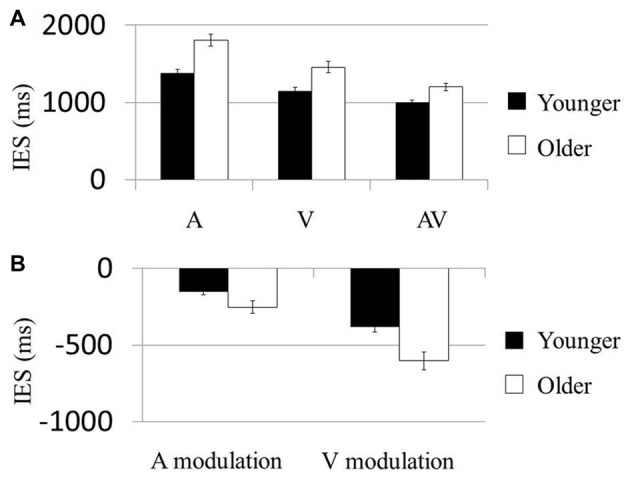
AV stimuli were more beneficial in older participants. **(A)** Mean inverse efficiency score (IES) in three conditions (A, V and AV) for younger and older participants. Younger participants performed better than the older participants in each condition. **(B)** “Modulation” scores were computed as the difference in IES between multisensory (AV) and unisensory (either A or V) conditions, allowing us to examine the degree by which AV information could improve performance in each subject. Participants who showed much greater behavioral improvements in the AV condition than in unisensory conditions were related to more negative benefit scores. The older group showed more negative A modulation and V modulation scores than the younger group. Error bars represent standard errors.

In the first model testing the condition-based performance, the Condition (A, V vs. AV) × Age (younger vs. older) effect on the IES was significant (*F*_(2,114)_ = 6.33, *P* = 0.007). The Condition (*F*_(2,114)_ = 101.68, *P* < 0.001; Figure 2A) and Age (*F*_(1,57)_ = 21.80, *P* < 0.001) effects were also significant. *Post hoc* analysis on the Condition effect showed that IES of the AV condition in both groups was significantly smaller than that in the A (Younger: *t*_(29)_ = 11.11, *P* < 0.001; Older: *t*_(28)_ = 10.71, *P* < 0.001) and V conditions (Younger: *t*_(29)_ = −33.86, *P* < 0.001; Older: *t*_(28)_ = −24.08, *P* < 0.001). These results suggest that each of the older and younger groups had faster normalized reaction times (i.e., IES) in the AV condition than in the A or V. *Post hoc* comparisons showed that younger participants had significantly faster normalized reaction times than older participants in all three conditions (A: *t*_(58)_ = −4.65, *P* < 0.001; V: *t*_(58)_ = −3.60, *P* = 0.001; AV: *t*_(58)_ = −3.65, *P* = 0.001). No result was presented for the control condition because the accuracy rate required to compute the IES was not a behavioral parameter captured in the task.

In the second model testing the multisensory modulation effect, the Modulation (AV-V vs. AV-A) × Age (younger vs. older) effect on IES was not significant (*F*_(1,57)_ = 2.65, *P* = 0.109; Figure 2B). However, the Modulation (*F*_(1,57)_ = 40.82, *P* < 0.001) and Age (*F*_(1,57)_ = 21.75, *P* < 0.001) effects were both significant. *Post hoc* analysis of the Age effect demonstrated that the modulations of participants’ performances in both the visual and the audial conditions were higher in the older group than the younger group (AV-V: *t*_(58)_ = −2.101, *P* < 0.042; AV-A: *t*_(58_) = −3.221, *P* < 0.002). Similarly, no result was presented for the control condition the accuracy rate required to compute the IES was not a behavioral parameter.

### ERP Results

#### P2 Consecutive Differences ~145 to 175 ms in Younger Group

**Figure 3:**
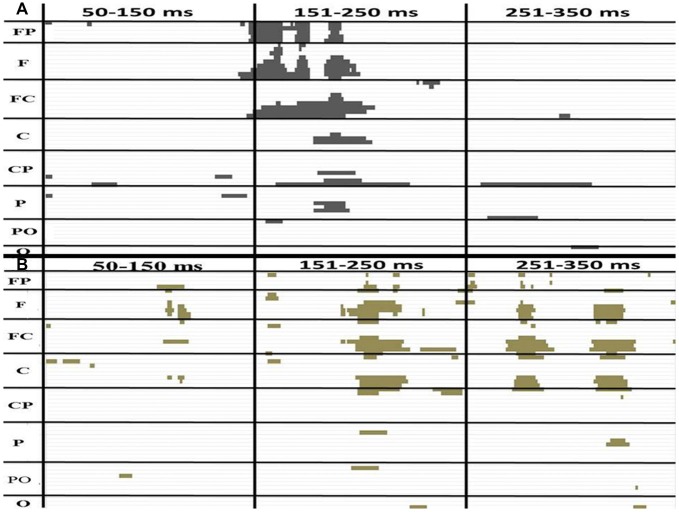
The *P*-value of a paired *t* test between (AV + C) and (A + V) in the younger **(A)** and older **(B)** groups. The *x* and *y* axes showed the timeline and electrodes, respectively. The figure only shows point at which the *P*-value is less than 0.05. The FP electrode group includes FP1, FPZ, FP2. The F group includes F7, F5, F3, F1, Fz, F2, F4, F6, F8. The FC group includes FT7, FC5, FC3, FC1, FCz, FC2, FC4, FC6, FT8. The C group includes T7, C5, C3, C1, Cz, C2, C4, C6, T8. The CP group includes TP7, CP5, CP3, CP1, CPz, CP2, CP4, CP6, TP8. The P group includes P7, P5, P3, P1, Pz, P2, P4, P6, P8. The PO group includes PO7, PO3, POz, PO4, PO8. The O group includes O1, Oz, O2.

**Figure 4:**
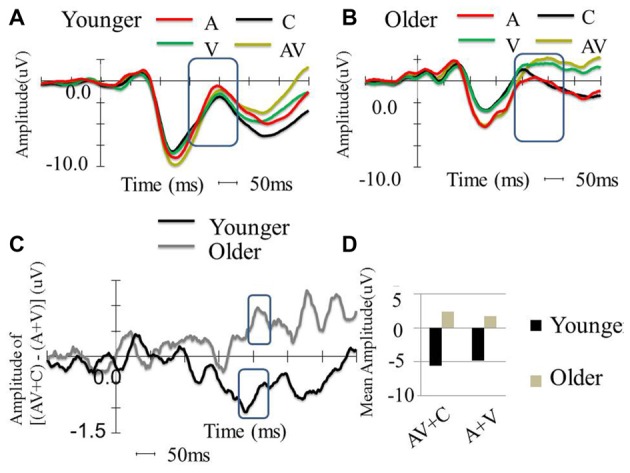
Between-group comparison of the event-related potential (ERP) waveform in the FC2 electrode. **(A)** ERP waveform of younger participants in four conditions (A, V, AV and C), which shows typical P1, N1 and P2. **(B)** Waveform of older participants in four conditions (A, V, AV and C), which shows typical P1, N1, and P2. **(C)** ERP amplitude differences of [(AV + C) − (A + V)] among the younger and older participants. The older participants show super-additive patterns, while the younger participants show sub-additive patterns. **(D)** P2 mean amplitude comparison between the two groups. The older participants showed more negative amplitude in all conditions compared to the younger participants. The older participants also showed more negative amplitude in the AV + C condition, while the younger participants showed more negative amplitude in the A + V condition.

The amplitudes of [(AV + C) − (A + V)] were significantly different from zero in the electrodes in the frontal (FPz), frontotemporal (F6 and F8) and fronto-central (FC4 and FC6) regions (Figure 3). The shapes of the waveforms in the A or V condition and the AV condition appeared to be similar (Figure 4C). The significant amplitudes of [(AV + C) − (A + V)] appeared to be due to the more positive-going (A + V) waveform when compared to the (AV + C). This pattern was observed throughout the entire epoch. The waveform in this time period appeared to correspond to the ascending limb of auditory P2 until just before its peak.

#### P2 Consecutive Differences ~175 to 220 ms in Younger Group

The amplitudes of [(AV + C) − (A + V)] significantly differed in the electrodes in the fronto-central (FC2, FC4 and FC6), central (C2 and C4) and temporo-parietal (TP8) regions (Figure 3). The waveforms of the A, V and AV conditions appeared to be similar (Figure 4C). The results of this time period appeared to correspond to the peak of the auditory P2.

#### P2 Consecutive Differences ~194 to 222 ms in Older Group

Unlike the younger participants, among the older participants only the ~194 to 222 ms consecutive time period significantly differed from zero (Figure 3). The amplitudes of [(AV + C) − (A + V)] were significant in the electrodes in the frontal (Fz), fronto-central (FC1 and FC) and central (C1) regions. The unisensory and multisensory waveforms were similar (Figure 3). The waveform patterns and differences among the conditions were similar to those for the younger group (Figure 4B). The waveform in this time period appeared to correspond to the ascending limb of P2 until its peak.

#### Younger and Older Group Comparisons

**Figure 5:**
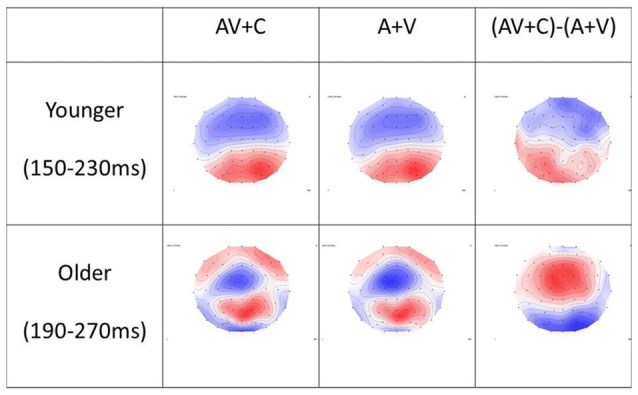
Topography of (A + V), (AV + C) and (AV + C) − (A + V) in younger and older groups. In the younger group, the time window of P2 was chosen as 150–230 ms after stimulus. The topography showed mainly negativity in the fronto-central region and positivity in the parieto-occipital region in all three conditions. In the older group, the time window of P2 was chosen as 190–270 ms after stimulus. Similar topography was observed in the (AV + C) and (A + V) conditions, while reversed topography was shown in the (AV + C) − (A + V) condition in relation to those for the younger group; that is, positivity was shown in the frontal-central region while negativity was shown in the parieto-occipital region, indicating a super-additive pattern in the fronto-central region.

The P2 showed significant differences in the peak latencies between the younger and older groups (A condition: *t*_(58)_ = −8.08, *P* < 0.001; V condition: *t*_(58)_ = −6.10, *P* < 0.001; AV condition: *t*_(58)_ = −9.32, *P* < 0.001). The older group demonstrated significantly more positive-going P2 amplitude than the younger group in the V and AV conditions (V condition: *t*_(58)_ = −4.61, *P* < 0.001; AV condition: *t*_(58)_ = −3.67, P *=* < 0.001) but not in the A condition (*t*_(58)_ = 1.609, *P* = 0.113). The P2 distribution was in the frontal-central region, and the electrode that showed significant difference in both groups between the two conditions in FC2 (Figures 4A, 5). The mean amplitude of P2 ([(AV + C) − (A + V)]) at FC2 was significantly more positive-going in the older group than the younger group (*t*_(58)_ = −3.31, *P* = 0.002). The significant P2 amplitude was significantly larger than zero in the older group (*t*_(30)_ = 2.63, *P* = 0.013), indicating super-additivity. In contrast, the significant P2 amplitude was significantly smaller than zero in the younger group (*t*_(29)_ = −2.15, *P* = 0.040), suggesting sub-additivity (Figure 4D and Table 1).

**Table 1:** Mean amplitude of P2 in four conditions (A, V, AV and C) for younger and older participants.

Mean amplitude/Condition	A	V	AV	C
Younger	−2.06 ± 4.59	−2.76 ± 3.53	−2.50 ± 4.66	−3.06 ± 3.66
Older	−0.21 ± 4.34	1.98 ± 4.36	2.00 ± 4.83	0.41 ± 3.51

*The figures are mean ± SD*.

#### Moderation Analysis with Behavioral Data

**Figure 6:**
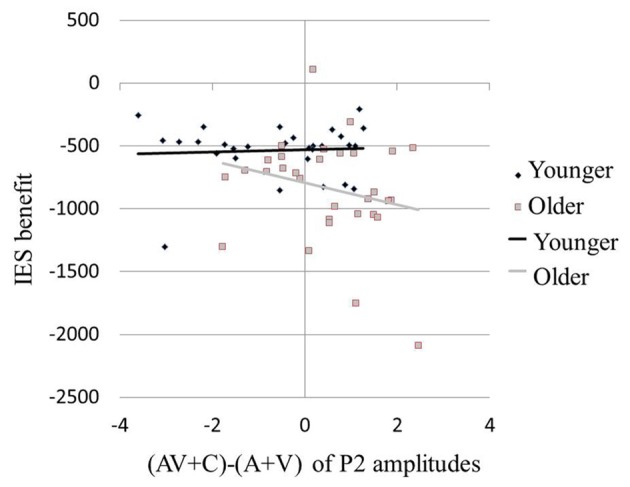
Larger behavioral benefits were related to greater neural integration in multisensory condition in older participants. Behavioral benefit in multisensory integration was indexed by the average of the V benefit [IES (AV-A)] and the A benefit [IES(AV-V)]. Neural integration of multisensory information was indexed by (AV + C) − (A + V) of P2 amplitudes in FC2. Older participants with larger behavioral benefits (more negative values) also showed greater super-additive neural integration (more positive values; squares). However, in younger participants, the degree in behavioral benefit was marginally related to the degree of neural integration (diamonds).

Results of the ERP revealed super-additive integration of auditory-visual information in older adults, but sub-additive integration in younger adults. The two-step regression model tested the relationships between the peak amplitudes [(AV + C) − (A + V)] of P2 and the modulation effects of the multisensory integration with age as a moderator. In the first step, Age (*β* = −0.331, *P* = 0.014) but not P2 amplitude (*β* = −0.174, *P* = 0.188) was a significant predictor of the IES scores of the participants, reflecting modulation by the multisensory integration, which accounted for 15.7% of the total variance (*F*_(2,59)_ = 6.45, *P* = 0.003). In the second step, the added Age × P2 amplitude regressor was significant and further improved the prediction of the participants’ IES scores for 16.2% of the total variance (Δ*R*^2^ = 0.162, Δ*F*_(1,59)_ = 9.94, *P* < 0.001). *Post hoc* analysis revealed that, in the younger group, the behavioral benefit score was marginally correlated with the P2 amplitude of (AV + C) − (A + V; *r* = 0.354, *P* = 0.060; Figure 6, diamonds). After deleting the outlier, no significant correlation between the behavioral benefit score and the P2 amplitude of (AV + C) − (A + V) was observed (*r* = −0.221, *P* = 0.258). In contrast, the behavioral benefit score was significantly correlated with the P2 amplitude of (AV + C) − (A + V; *r* = −0.423, *P* = 0.018; Figure 6, squares) in the older group.

The mean total score of MoCA was 27.3 for the older participants. The four subscales that displayed relatively lower scores were memory (mean = 3.4 out of 5), abstraction (mean = 1.7 out of 2), attention (mean = 4.6 out of 5), and visuospatial/executive (mean = 4.7 out of 5). These score were submitted to correlation analyses with the EEG amplitudes. No significant correlations were revealed between the total score of MoCA and the amplitudes of P2 for the AV, A and V conditions or [(AV + C) − (A + V)] (*P* > 0.050). Significant correlations were yielded between the attention subscale score on MoCA with [(AV + C) − (A + V)] (*r* = 0.37, *P* = 0.043) and the V condition (*r* = −0.42, *P* = 0.020). No other correlations were statistically significant for [(AV + C) − (A + V)] (*P* > 0.050).

## Discussion

This study aimed to investigate the impact of aging on the AV integration process. The results indicated that the age-related effect modulated the integrative process in both the perceptual and feedback stages. Specifically, the significant multisensory P2 elicited in the central and fronto-central regions revealed that super-additive processes were likely to occur in the older group but not in the younger group. The super-additive process was indicated by significantly higher [AV + C] than [A + V] amplitudes. The findings are corroborated with larger improvements in the older group than the younger group in their performances on the spatial discrimination task. The non-significant results obtained for the occipital region in the older group suggested that multisensory integration appeared to exert stronger modulatory effects on auditory rather than visual functions. The modulation effect among older participants may serve the role of compensating for their relatively declined cognitive abilities, such as attention function.

The sub-additive pattern of the fronto-central P2 found in the younger group is consistent with those reported in Stekelenburg and Vroomen (2007), which demonstrated that healthy young participants showed decreased P2 amplitude in AV compared to unisensory conditions. The results of previous studies of the aging effect on the fronto-central P2 render a plausible explanation for the delayed latency and super-additive pattern revealed in the older group. Fronto-central P2 is a common aging-effect marker evoked by different tasks. For instance, differences in the amplitude of fronto-central P2 were related to the aging effect on the allocation of resources in attentional tasks (Moreno et al., 2011; Wild-Wall et al., 2012; Staub et al., 2014; Tallus et al., 2015) and the aging effect on evaluation of stimulus in response-conflict tasks (Potts, 2004; Gajewski et al., 2008). The significant delay in the P2 latency over the fronto-central area revealed in the older group suggested that the older participants might experience greater delays in the auditory discrimination process when compared to their younger counterparts (Lister et al., 2011). On the other hand, the significant increases in the P2 amplitude of [(AV + C) − (A + V)], i.e., the super-additive pattern, indicated that the age-related effect is likely to act on the stimulus-evaluation process among older participants (Gajewski et al., 2008).

The task employed in this study involved encoding and discrimination of combined auditory and visual stimuli. Participants were asked to discriminate four locations with the visuospatial information conveyed from the stimuli. Significantly higher fronto-central P2 amplitudes in the older group than the younger group in the AV condition reflected that evaluation of the combined stimuli—making use of the integrated visuospatial information—was crucial for participants to give the appropriate visuospatial responses. This proposition is further supported by the finding that the super-additive pattern of P2 in the AV condition predicted spatial discrimination task performance for the older group but not the younger group. Getzmann et al. (2013) reported further evidence that corroborates our findings, demonstrating that providing training in pitch discrimination with background noise to older participants improved their performances on the task and yielded stronger P2 amplitudes (Getzmann et al., 2013). A recent study on auditory discrimination reported that fronto-central P2 was related to judgment of the auditory frequency process (Noguchi et al., 2015). One could argue that older participants’ lower performances on the spatial discrimination task in this study was due to their deteriorated audial and visual senses. Calibration of the difficulty levels of audial and visual trials in the A, V and AV conditions and the training provided to both the older and younger participants were useful for ruling out this possibility. Altogether, the super-additive pattern of P2 observed in the older participants indicates that the AV effect is likely attributable to the enhanced encoding and evaluation of the auditory stimuli, which may deteriorate due to the aging effect.

The super-additive pattern of P2 observed among the older participants can also be related to the aging effect on attention function. Community-dwelling older adults were reported to experience a general decline in cognitive functions particularly in attention, working memory and executive functions (Hsu et al., 2015; Chan et al., 2017). Fronto-centrally distributed P2 has been suggested to be marker of attention, particularly in relation to physical properties of sound (e.g., Tiitinen et al., 2006). For the older participants in this study, the amplitudes of the AV integrative P2, i.e., [(AV + C) − (A + V)], were only significantly correlated with their attention subscale scores of MoCA. In other words, older participants who had higher attention ability gained higher levels of the super-additive modulation for enhancing spatial discrimination. On the other hand, those who had lower attention ability gained less from the AV integration. Our findings concur with those reported in other studies that decreased attention function was significantly involved in both multimodal (Alain and Woods, 1999; Poliakoff et al., 2006) and unimodal conditions (Andrés et al., 2006; Yang and Hasher, 2007) among older participants. Our observation on the attention function was further corroborated with the finding that the super-additive pattern of P2 in the older participants was found to significantly predict their performance on the AV condition in terms of normalized reaction time.

Our results are different from those reported by Stephen et al. (2010), who employed MEG on multisensory integration. The age-related differences in the 200 ms post-stimulus time-window were in the temporal region (STG, superior temporal gurus). Other brain imaging studies reported the STG as a heteromodal cortex for auditory and visual information (e.g., Baumann and Greenlee, 2007). It is plausible that the inconsistent results are attributable to inclusion of the “noise” condition in this study in computation of the multisensory effect of [(AV + C) − (A + V)] when compared with that of AV-A used by Stephen et al. (2010). Additional analysis conducted on the AV-A comparison showed a consistent super-additive pattern in the older group (*t*_(30)_ = −6.61, *P* < 0.001) and sub-additive pattern in the younger group (*t*_(28)_ = 2.12, *P* = 0.043). It is noteworthy that in this study the use of (AV—V) and (AV—A) for analyzing the between-group differences in the gains due to the AV integration is deemed useful. The adjustment of the potential baseline differences in the A and V conditions between younger and older participants as revealed from the second ANOVA model yielded more unbiased comparisons than the conventional A, V and AV comparisons as revealed from the first ANOVA model. Inclusion of the control condition has the advantage of partially contrasting the perceptual processes associated with the unisensory visual and auditory stimuli, hence the signal-to-noise ratio of the multisensory integration processes is increased (Talsma and Woldorff, 2005; Gondan and Röder, 2006; Mishra et al., 2007; von Saldern and Noppeney, 2013). Nevertheless, this proposition needs to be further studied by employing simultaneous EEG and functional imaging methods. Another inconsistent finding is that no significant results were revealed in the current study for the early components, such as C1 and P1 (around 100 ms post stimulus). This could have been due to the noise added to the audial and visual stimuli, which was not a factor in Stephen’s study. A recent fMRI study that employed a similar design with noise embedded in the stimuli reported non-significant activations in the primary sensory regions, which mediated the perceptual processes (von Saldern and Noppeney, 2013). As the early components such as C1 reflect processes in the primary sensory cortices (Cappe et al., 2010), future studies should explore plausible explanations for this phenomenon.

In this study, a between-group difference in the multisensory integration modulation was observed in the fronto-central but not in the occipital-parietal P2. It is plausible that the results could have been confounded by the visual-to-auditory biases in the perceptual processes during the task. Evidence suggests that visual information is generally more informative than auditory information in spatial discrimination (Beierholm et al., 2009; Callan et al., 2015). As a result, the participants, particularly those in the older group, could have relied more on visual than auditory information during the multisensory condition. This proposition is less convincing because the difficulty levels of the visual and auditory stimuli were adjusted in the construction of the task, and the older and younger participants’ performances on the unisensory conditions were not significantly different after the training. Another possible explanation for the fronto-central, but not occipital-parietal, modulation is that the older participants had genuine reliance on visual information for processing. Our results concur with a previous study on speech perception that found that older participants relied more on visual than auditory input, while the reverse was true for younger participants (Cienkowski and Carney, 2002). The reliance on visual rather than auditory input among older adults was due to the decreased sensitivity of their peripheral and central auditory system (Freigang et al., 2014). Recent studies further reported optimal binding of information from different modalities was not innate but rather learned gradually from experience (Bauer et al., 2015; Hecht and Gepperth, 2015). Greater reliance on visual rather than auditory stimuli was evident from the significantly higher accuracy rates in the unisensory visual condition compared to the auditory condition in the spatial discrimination task in the older group (*P* = 0.005) but not in the younger group (*P* = 0.834). This offers a plausible explanation for the multisensory modulation effect observed in the fronto-central but not in the occipito-parietal P2. Therefore, the modality on which older participants relied more (the visual modality) could help the other modality to achieve a greater ability for stimulus evaluation, further improving their behavioral performance.

### Limitations

This study demonstrated that age can modulate multisensory integrative processes in the perceptual and feedback stages. The use of the wrist movement response device could produce unnecessary artifacts that may have contaminated the results. Future studies should explore other response devices that can produce fewer artifacts and hence enhance the quality of the data. The findings on multisensory integration are based on a spatial discrimination task relying on audial and visual stimuli. The results obtained are limited to these two modalities and the integrative process in spatial discrimination; therefore, caution should be taken not to generalize these results to other sensory modalities and integrative processes. Future studies will extend to other modalities, such as haptic sense, and other AV processes, such as spatial localization. The model adopted to explain AV integration in this study was [(AV + C) − (A + V)]. Interpretation of the results is bound by the model, particularly the linear relationships between the multisensory and unisensory factors. The validity of this model should be further tested by incorporating other comparative models on the aging effect. Because of the high difficulty level of the task, participants older than 75 years were not included in the study; this would have limited the generalization of the results to individuals older than 75 years old. This study focused on the perceptual process (50–350 ms) of AV stimuli. Further studies will explore how the modulated perceptual process could affect subsequent processing of the integrated signals—for example, in motor and emotional processes. Last but not least is the uncorrected p-level set for the correlation yielded between AV integrative P2 and attention function, our proposition on the role of AV integration in compensating attention function among older adults should be further tested in future study.

## Conclusion

The results of this study revealed the dissociation of the multisensory integrative process between younger and older participants. The aging effect modulating the AV integrative processes in spatial discrimination was found to occur in the perceptual and feedback stages. This was reflected by the significant fronto-centrally distributed P2, suggesting that super-additivity of the auditory and visual information occurred in the older group but not in the younger group. The AV gains due to the super-additive P2, however, appeared to be attributable to the auditory rather than the visual information. The super-additive P2 was also found to be significantly related to the attention function of participants in the older group. This finding suggests that AV integration may serve a functional role to compensate for the deterioration of attention function, due to aging, during the spatial discrimination process.

## Author Contributions

ZZ: conceptualization, task design, data collection, data analysis, interpretation of data and writing the article. BKHC: conceptualization, data analysis, interpretation of data and writing the article. K-HT: conceptualization, method, data analysis, interpretation of data and writing the article. CCHC: conceptualization, task design, interpretation of data and writing the article.

## Conflict of Interest Statement

The authors declare that the research was conducted in the absence of any commercial or financial relationships that could be construed as a potential conflict of interest.
